# Depressive symptoms increase the risk of falls and injurious falls in Chinese adults aged ≥ 45 years: A 3-year cohort study

**DOI:** 10.3389/fpubh.2022.964408

**Published:** 2022-10-13

**Authors:** Hanli Bu, Suqing Lu, Linxian Wang, Dan Jiang, Zhenzhen Tian, Yi Ding, Qin Zhuang

**Affiliations:** ^1^Department of General Practice, Affiliated Hospital of Jiangsu University, Zhenjiang, China; ^2^Department of Ophthalmology, Affiliated Hospital of Guilin Medical University, Guilin, China

**Keywords:** depressive symptoms, fall, injurious fall, cohort study (or longitudinal study), CHARLS

## Abstract

**Background:**

Falls and depressive symptoms are both public health concerns in China, but the effects of depressive symptoms on falls and injurious falls have not been thoroughly investigated.

**Methods:**

This population-based prospective cohort study used data derived from adults aged ≥45 years acquired from the 2015 and 2018 China Health and Retirement Longitudinal Study. Data were analyzed from August 2021 to December 2021. Self-reported depressive symptoms were determined using a 10-item Center for Epidemiologic Studies Depression scale (CESD-10) with a total score range of 0–30. Item responses of 3–4 or 5–7 days were deemed indicative of specific depressive symptoms. The outcome variables were self-reported accidental falls and injurious falls.

**Results:**

Of the 12,392 participants included in the study, 3,671 (29.6%) had high baseline depressive symptoms (CESD-10 scores ≥ 10), 1,892 (15.3%) experienced falls, and 805 (6.5%) experienced injurious falls during 2015–2018 follow-up. High depressive symptoms increased the risk of falls [odds ratio (OR) 1.34, 95% confidence interval (CI) 1.19–1.50] and injurious falls (OR 1.28, 95% CI 1.09–1.51) in a multivariable logistic regression model adjusted for major demographic, health-related, and anthropometric covariates. All of the 10 specific depressive symptoms except “felt hopeless” were associated with falls, and four specific symptoms significantly increased the risk of injurious falls; “had trouble concentrating” (OR 1.32, 95% CI 1.13–1.55); “felt depressed” (OR 1.32, 95% CI 1.12–1.55); “everything was an effort” (OR 1.23, 95% CI 1.04–1.45); and “restless sleep” (OR 1.18, 95% CI 1.02–1.40).

**Conclusion:**

High depressive symptoms are significantly related to risk of falls and injurious falls. Four specific symptoms (had trouble concentrating, felt depressed, everything was an effort, and restless sleep) increase the risk of injurious falls in Chinese adults aged ≥ 45 years.

## Introduction

As populations age, falls are becoming an increasing global health concern (1). Injuries resulting from severe falls such as fractures and traumatic brain injuries require medical treatment. These injuries can lead to chronic pain, disability, and even death (2, 3). Worldwide 37.3 million patients who suffer from falls require medical treatment each year, and falls cause the loss of >17 million disability-adjusted life-years (1). In 2019 the morbidity of falls in mainland China in adults aged >60 years was 3,799.4 per 100,000. The incidence rate of deaths associated with falls was 39.2 per 100,000, and that of disability-adjusted life-years associated with falls was 1,238.9 per 100,000 (4). Detecting, modifying, and preventing risk factors for falls in adults may reduce the number of falls, injurious falls, and associated complications.

Depressive symptoms are a mental problem characterized by sadness, loss of interest in activities, and reduced energy (5). They are typically measured by validated rating scales with established cutoffs such as the Epidemiologic Studies Depression scale (6). Depressive symptoms are consistently associated with falls (7–11). Two studies conducted in the United States and Canada demonstrated significant associations between baseline depressive symptoms and subsequent falls in community-dwelling older populations (7, 9). Systematic reviews (10, 11) also indicate a relationship between high depressive symptoms and an increased risk of falls in older adults. Notably, previous studies (7–11) have considered depressive symptoms as a dichotomous factor (e.g., present or not), or have used a total depression score. Such studies assumed that depressive symptoms are a single condition, and that all specific symptoms are fungible and equally valid indicators. Specific depressive symptoms such as sadness, sleep disorders, inattention, and suicidal ideation are distinct conditions, and they differ in significant respects such as potential biology, effect on injury, and risk factors (12). Moreover, most prior studies have concentrated on the role of depressive symptoms only on the occurrence of falls, while ignoring resulting injuries that required medical care (8–11), or they have only examined these relationships in older people (9–11).

The aim of the current study was to investigate associations between depressive symptoms—including elevated high depressive symptoms and specific depressive symptoms—and falls and injurious falls in Chinese populations aged 45 years and over.

## Materials and methods

### Study design

This study was a secondary analysis of data from the 2015 and 2018 China Health and Retirement Longitudinal Study (CHARLS). The CHARLS was a longitudinal survey-based study that aimed to be representative of residents aged 45 years and over in mainland China (13). It covered 28 provinces, 150 counties, and 450 villages/urban communities. A stratified (by per capita gross domestic product of urban districts and rural counties) multistage (county/district-village/community household) probability proportional to population size random sampling strategy was used. The first national survey was conducted in 2011, and the most recent was conducted in 2018. Through face-to-face interviews using a standardized questionnaire, detailed demographic and health-related data were acquired. The CHARLS was confirmed by the Biomedical Ethics Review Committee of Peking University, Beijing, China (IRB00001052–11015) (13).

### Study participants

**Figure 1:**
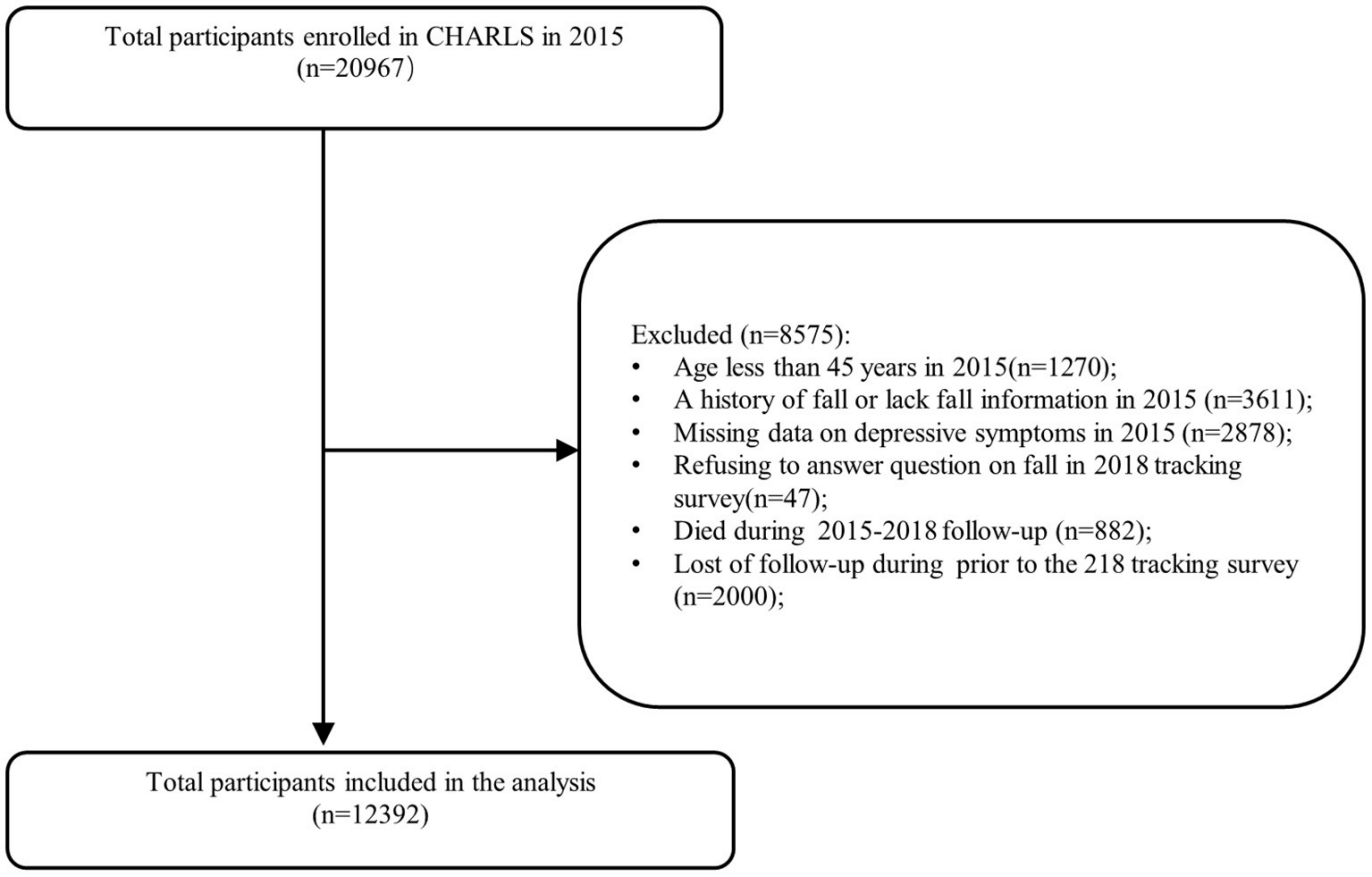
Flow chart of the participants.

Participants who were included in both the 2015 cohort and the subsequent 2018 tracking survey were considered for inclusion in the present study. The exclusion criteria were (1) age <45 years; (2) a history of falls or a lack of fall information in 2015; (3) missing data on depressive symptoms in 2015; (4) refusing to answer questions on falls in the 2018 tracking survey; (5) died during the 2015–2018 follow-up period; and (6) lost to follow-up prior to the 2018 tracking survey. The final sample analyzed included 12,392 individuals (Figure 1).

### Depressive symptoms

The Center for Epidemiologic Studies Depression scale comprised of ten items (CESD-10) in the CHARLS was used to measure depressive symptoms (13). Based on the number of days participants had experienced depressive symptoms in the past week, items were rated as < 1 day (0 points), 1–2 days (1 point), 3–4 days (2 points), or 5–7 days (3 points). The total CESD-10 score was the sum of all items, and possible scores ranged from 0 to 30. A CESD-10 score ≥ 10 was considered indicative of significant depressive symptoms (14). Participants with high depressive symptoms were divided into two subgroups; moderate (9–19) and higher (20–29). Using the method described by Jokela et al. (15), item responses of 3–4 or 5–7 days were deemed indicative of specific depressive symptoms.

### Falls and injurious falls

In the survey falls were alluded to with the question “Have you fallen down?”. Possible answers were “yes” or “no.” If the answer was “yes,” participants were asked a subsequent question, “How many times have you fallen down seriously enough to need medical treatment?”. Participants who reported needing medical treatment were defined as having had injurious falls. Outcome variables were treated as binary.

### Assessment of covariates

Demographic information including age, gender, residence, marriage, and education were obtained *via* a structured questionnaire. Physician diagnoses of chronic comorbidities, including hypertension, diabetes, chronic lung diseases, heart diseases, kidney diseases, neuropsychiatric diseases, and arthritis or rheumatism were self-reported. Neuropsychiatric diseases were defined as the combination of stroke, emotional or mental problems, and memory-related diseases. Other health-related covariates included were drinking habits, smoking habits, body pains, and medication use.

Measurements of anthropometric indicators including systolic blood pressure, diastolic blood pressure, pulse, body mass index, waist circumference, handgrip strength, and peak expiratory flow were performed by trained research nurses. Handgrip strength was defined as the maximum strength of the dominant hand. Blood samples were provided by 68.5% (8,487/12,392) of the participants, and were used to assess white blood cells, platelets, hemoglobin, C-reactive protein, creatinine, glucose, and uric acid. Glomerular filtration rate was estimated using the Chronic Kidney Disease Epidemiology Collaboration's 2009 creatinine equation (16).

### Statistical analysis

Data were analyzed from August 2021 to November 2021. Continuous variables are represented as means and standard deviations, or medians and interquartile ranges. Categorical variables are represented as frequencies and percentages. Differences between groups were assessed using the *t*-test, χ^2^ test, analysis of variance, or Mann–Whitney *U*-test. There was 23.1% (2,866/12,392) of baseline data missing, and these data were imputed using the R-package missForest.

Multivariate logistic regression analyses were performed to identify odds ratios (ORs) and 95% confidence intervals (CIs). An extended model approach was used for covariate adjustment, where model 1 was adjusted for age and gender; model 2 was adjusted for age, gender, residence, marriage, education, smoking, and drinking; model 3 was adjusted for model 2 and included body pains, history of hypertension, diabetes, chronic lung diseases, heart diseases, kidney diseases, neuropsychiatric diseases, arthritis or rheumatism, hypertension medications, diabetes medications, heart medications, and neuropsychiatric medications; and model 4 was adjusted for model 3 and included systolic blood pressure, diastolic blood pressure, pulse, body mass index, waist circumference, handgrip strength, and peak expiratory flow. Potential nonlinear associations were also investigated *via* restricted cubic spine regression.

*Post-hoc* subgroup analyses were performed to investigate associations between some baseline characteristics and falls and injurious falls. *p*-values for interactions were determined *via* likelihood ratio tests. Sensitivity analyses were performed to evaluate the robustness of results. First, further adjustment for laboratory indicators in model 4 was conducted in 8,538 participants who provided blood samples. Analyses were then repeated in 9,526 participants with non-imputed data.

Variance inflation factors with a cutoff value of 10 were generated to assess multicollinearity of the variables in the models. All analyses were performed in Stata version 16 and R version 4.1.1, and two-tailed *p*-values < 0.05 were considered statistically significant.

## Results

### Study participants and characteristics

The mean age of the 12, 392 study population was 58.0 years, 50.6% were men, and 29.6% had high depressive symptoms (CESD-10 score ≥ 10). Compared with those with low depressive symptoms (CESD-10 score < 10), participants with high depressive symptoms were less likely to be male, younger, urban residents, and married. They were more likely to have no formal education, be nonsmokers and nondrinkers, and have body pains. Participants with high depressive symptoms also had a greater percentage of self-reported physician-diagnosed medical conditions and medication use, lower systolic and diastolic blood pressure, lower body mass index, smaller waist circumference, weaker handgrip strength, lower peak expiratory flow, and lower levels of white blood cells, hemoglobin, C-reactive protein, glucose, estimated glomerular filtration rate, and uric acid (Table 1). Baseline differences between included and excluded individuals are shown in Supplementary Table 1.

**Table 1:** Baseline characteristics of 12,392 participants by depressive symptoms.

	**Participants, no. (%)**			
	**Total sample**	**Depressive symptoms**		
**Characteristics**	**(*N* = 12,392)**	**Low, <10 (*N* = 8,721)**	**High, ≥10 (*N* = 3,671)**	***P*-value^a^**
Age, y	58.00 (51.00, 65.00)	58.00 (51.00, 65.00)	59.00 (52.00, 66.00)	<0.001
Man	6,266 (50.56)	4,815 (55.21)	1,451 (39.53)	<0.001
Rural residence	4,814 (38.85)	3,682 (42.22)	1,132 (30.84)	<0.001
Married	10,367 (83.66)	7,476 (85.72)	2,891 (78.75)	<0.001
**Educational level**				<0.001
No formal education	4,675 (37.73)	2,884 (33.07)	1,791 (48.79)	
Primary school	3,577 (28.87)	2,597 (29.78)	980 (26.70)	
Middle or high school	3,646 (29.42)	2,820 (32.34)	826 (22.50)	
College or above	494 (3.99)	420 (4.82)	74 (2.02)	
Smoke^b^	5,571 (44.96)	4,142 (47.50)	1,429 (38.93)	<0.001
Drink^b^	5,826 (47.05)	4,294 (49.27)	1,532 (41.77)	<0.001
Body pains^b^	3,178 (25.65)	1,325 (15.20)	1,853 (50.48)	<0.001
**History of comorbidities**				
Hypertension^b^	3,754 (30.45)	2,506 (28.87)	1,248 (34.19)	<0.001
Diabetes^b^	973 (7.91)	624 (7.20)	349 (9.61)	<0.001
Chronic lung diseases^b^	1,524 (12.36)	869 (10.01)	655 (17.97)	<0.001
Heart diseases^b^	1,661 (13.48)	1,016 (11.71)	645 (17.71)	<0.001
Kidney diseases^b^	714 (5.80)	390 (4.49)	324 (8.90)	<0.001
Neuropsychiatric diseases^b^	558 (4.52)	261 (3.00)	297 (8.13)	<0.001
Arthritis or rheumatism^b^	3,897 (31.58)	2,246 (25.86)	1,651 (45.17)	<0.001
**History of medication use**				
Hypertension medications^b^	2,806 (22.92)	1,848 (21.41)	958 (26.54)	<0.001
Diabetes medications^b^	675 (5.49)	433 (5.00)	242 (6.66)	<0.001
Heart medications^b^	1,069 (8.69)	618 (7.13)	451 (12.41)	<0.001
Neuropsychiatric medications^b^	250 (2.06)	117 (1.36)	133 (3.78)	<0.001
Systolic blood pressure, mmHg^b^	127.00 (19.21)	127.36 (19.02)	126.17 (19.60)	0.005
Diastolic blood pressure, mmHg^b^	75.15 (11.14)	75.46 (11.08)	74.44 (11.25)	<0.001
Pulse, /min^b^	73.83 (10.46)	73.85 (10.49)	73.79 (10.40)	0.813
Body mass index, kg/m^2b^	24.01 (3.64)	24.15 (3.62)	23.70 (3.69)	<0.001
Waist circumference, cm^b^	85.79 (12.69)	86.36 (12.49)	84.48 (13.05)	<0.001
Handgrip strength, kg^b^	30.98 (10.50)	32.24 (10.40)	28.06 (10.14)	<0.001
Peak expiratory flow, l/min^b^	327.79 (122.77)	339.59 (124.13)	300.51 (115.05)	<0.001
**Laboratory indictors** ^**c**^				
White blood cell, 10^9^/L	5.95 (1.66)	5.98 (1.64)	5.89 (1.69)	0.023
Hemoglobin, g/dL	13.77 (1.79)	13.88 (1.79)	13.53 (1.78)	<0.001
Platelet, 10^9^/L	203.42 (66.06)	203.01 (65.20)	204.37 (68.02)	0.393
C-reactive protein, mg/L	1.40 (0.70, 2.60)	1.40 (0.70, 2.50)	1.40 (0.80, 2.70)	0.023
Estimated glomerular filtration rate, mL/min/1.73 m^2^	99.32 (17.14)	100.17 (17.27)	97.34 (16.68)	<0.001
Glucose, mg/dL	95.50 (88.29, 106.31)	95.50 (88.29, 106.31)	95.50 (86.49, 106.31)	0.037
Uric acid, mg/dL	4.95 (1.36)	5.03 (1.37)	4.77 (1.31)	<0.001

Data are shown as means ± standard deviation, median (interquartile range), or numbers (percentages).

a *P*-value was based on χ^2^ or analysis of variance or Mann-Whitney *U*-test where appropriate.

b Missing data: 1 (0.01%) for smoke, 9 (0.07%) for drink, 3 (0.02%) for body pains, 63 (0.50%) for hypertension, 94 (0.76%) for diabetes, 64 (0.52%) for chronic lung diseases, 72 (0.58%) for heart diseases, 74 (0.60%) for kidney diseases, 54 (0.44%) for neuropsychiatric diseases, 51 (0.41%) for arthritis or rheumatism, 152 (1.23%) for hypertension medications, 105 (0.85%) for diabetes medications, 87 (0.70%) for heart medications, 273 (2.20%) for neuropsychiatric medications, 2,165 (17.47%) for systolic blood pressure, 2,167 (17.49%) for diastolic blood pressure, 2,170 (17.51%) for pulse, 2,115 (17.07%) for body mass index, 2,109 (17.02%) for waist circumference,2,170 (17.51%) for handgrip strength and 2,197 (17.73%) for peak expiratory flow.

c Measured in subpopulation of 8,487 (68.50%) participants.

### Associations between depressive symptoms and falls and injurious falls

**Figure 2:**
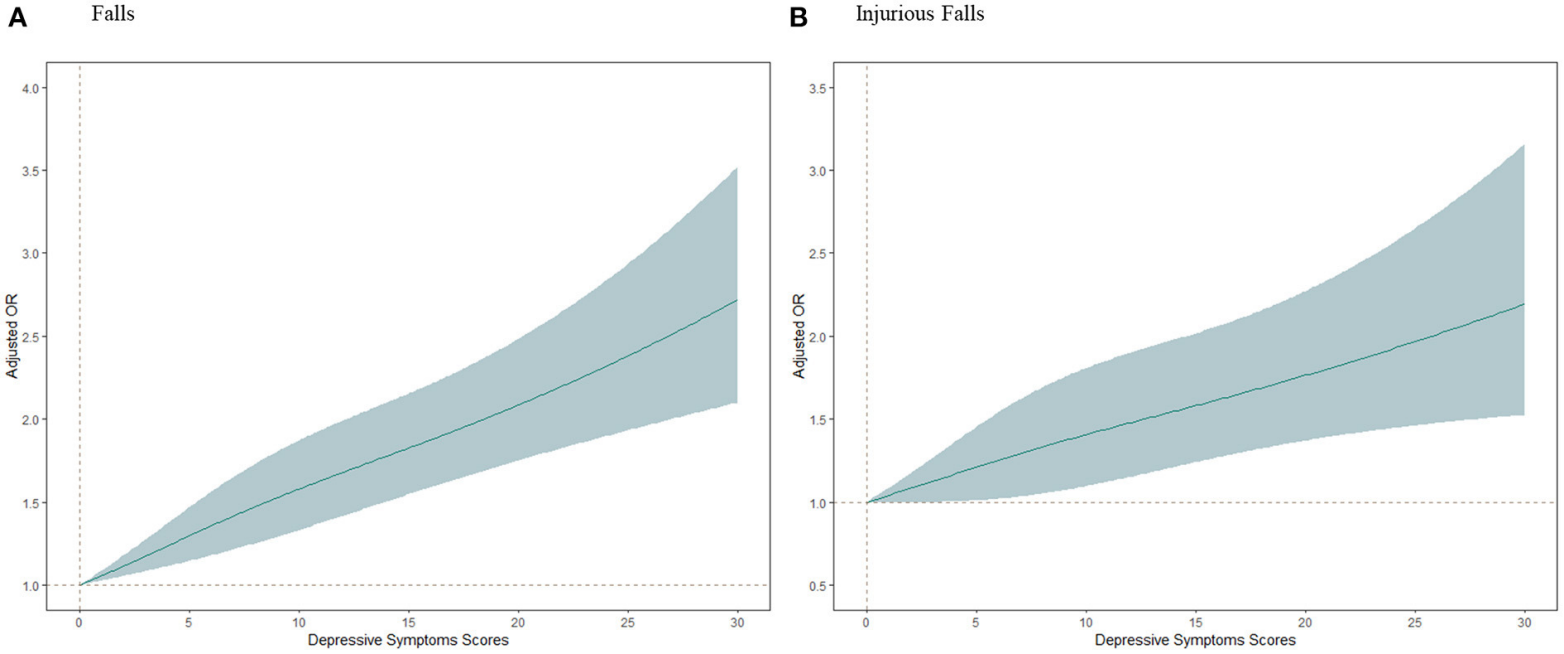
Adjusted odds ratios (ORs) of falls and injurious falls according to depressive symptoms scores. Graphs show ORs for falls **(A)** and injurious falls **(B)** adjusted for age, gender, residence, marital status, educational level, smoking status, drinking status, body pains, history of hypertension, diabetes, chronic lung diseases, heart diseases, kidney diseases, neuropsychiatric diseases and arthritis or rheumatism; and use hypertension medications, diabetes medications, heart medications, neuropsychiatric medications; and systolic blood pressure, diastolic blood pressure, pulse, body mass index, waist circumference, handgrip strength and peak expiratory flow. Data were fitted by a restricted cubic spline generalized linear regression model. The depressive symptoms score ranges from 0 to 30, with the highest score representing the highest risk of depressive symptoms. Solid lines indicate ORs, and shaded part indicate 95% confidence intervals (CI).

During the follow-up period from August 2015 to August 2018, a total of 1,892 participants experienced falls, and 805 of those had injurious falls. The incidence rates of falls and injurious falls were higher in individuals with high depressive symptoms. High depressive symptoms independently predicted the occurrence of falls (OR 1.34, 95% CI 1.19–1.50) and injurious falls (OR 1.28, 95% CI 1.09–1.51) after adjustments for all covariates. These associations remained significant when depressive symptoms were divided into three categories (Table 2). All of the specific depressive symptoms except “feel hopeless” were significantly associated with falls, and four of the specific symptoms were significantly associated with injurious falls; “had trouble concentrating” (OR 1.32, 95% CI 1.13–1.55), “felt depressed” (OR 1.32, 95% CI 1.12–1.55), “everything was an effort” (OR 1.24, 95% CI 1.04–1.45), and “restless sleep” (R 1.20, 95% CI 1.02–1.40) (Table 3). In restricted cubic spine regression analysis. there were linear positive associations between total CESD-10 score and the risks of falls and injurious falls (nonlinearity; *p* = 0.15 for falls, *p* = 0.54 for injurious falls) (Figure 2).

**Table 2:** The association between depressive symptoms and falls and injurious falls in 3-year follow-up (2015–2018).

**Depressive symptoms**	**Event**	**Incidence rate,** **per 1,000 person-years**	**OR (95%CI)**			
			**Model 1^a^**	**Model 2^b^**	**Model 3^c^**	**Model 4^d^**
**Falls**	1,892	50.89				
**Categories**						
Low, <10	1,140	43.57	1 [Reference]	1 [Reference]	1 [Reference]	1 [Reference]
High, ≥10	752	68.28	1.62 (1.46–1.80)	1.58 (1.42–1.76)	1.37 (1.22–1.53)	1.34 (1.19–1.50)
**Categories**						
Low, <10	1,140	43.57	1 [Reference]	1 [Reference]	1 [Reference]	1 [Reference]
Moderate, 10 to <21	576	63.43	1.49 (1.34–1.67)	1.46 (1.31–1.64)	1.30 (1.15–1.46)	1.27 (1.13–1.43)
Higher, ≥21	176	91.10	2.29 (1.90–2.76)	2.22 (1.84–2.68)	1.79 (1.46–2.18)	1.73 (1.41–2.11)
*p* for trend			<0.001	<0.001	<0.001	<0.001
**Injurious Falls**	805	21.65				
**Categories**						
Low, <10	475	18.16	1 [Reference]	1 [Reference]	1 [Reference]	1 [Reference]
High, ≥10	330	29.96	1.59 (1.37–1.85)	1.51 (1.30–1.76)	1.32 (1.12–1.55)	1.28 (1.09–1.51)
**Categories**						
Low, <10	475	18.16	1 [Reference]	1 [Reference]	1 [Reference]	1 [Reference]
Moderate, 10 to <21	252	27.75	1.48 (1.26–1.74)	1.42 (1.20–1.67)	1.26 (1.06–1.50)	1.23 (1.03–1.46)
Higher, ≥21	78	40.37	2.12 (1.63–2.73)	1.97 (1.51–2.55)	1.64 (1.23–2.15)	1.55 (1.16–2.03)
*p* for trend			<0.001	<0.001	<0.001	<0.001

OR, odds ratio; CI, confidence interval.

a Model 1 was adjusted for age and gender.

b Model 2 was adjusted for age, gender, residence, marital status, educational level, smoking status, drinking status.

c Model 3 was adjusted as model 2 plus body pains, history of hypertension, diabetes, chronic lung diseases, heart diseases, kidney diseases, neuropsychiatric diseases and arthritis or rheumatism; and use hypertension medications, diabetes medications, heart medications, neuropsychiatric medications.

d Model 4 was adjusted as model 3 plus systolic blood pressure, diastolic blood pressure, pulse, body mass index, waist circumference, handgrip strength and peak expiratory flow.

**Table 3:** Association between specific depressive symptoms and falls and injurious falls.

	**Event (%)**	**OR (95% CI)** ^**a**^	
		**Falls**	**Injurious falls**
Depressive symptoms number^b^		1.08 (1.05–1.10)	1.06 (1.03–1.10)
**Items** ^**c**^			
Bothered by little things	3,237 (26.12)	1.22 (1.09–1.37)	1.11 (0.94–1.30)
Had trouble concentrating	3,149 (25.41)	1.31 (1.17–1.46)	1.32 (1.13–1.55)
Felt depressed	3,113 (25.12)	1.34 (1.20–1.50)	1.32 (1.12–1.55)
Everything was an effort	3,023 (24.39)	1.26 (1.12–1.41)	1.23 (1.04–1.45)
Felt hopeless	5,339 (43.08)	1.06 (0.96–1.17)	1.02 (0.88–1.18)
Felt fearful	1,025 (8.27)	1.31 (1.11–1.54)	1.12 (0.88–1.41)
Restless sleep	3,854 (31.10)	1.15 (1.03–1.28)	1.20 (1.02–1.40)
Felt unhappy	3,810 (30.75)	1.18 (1.06–1.31)	1.11 (0.95–1.29)
Felt lonely	1817 (14.66)	1.21 (1.05–1.38)	1.17 (0.96–1.41)
Could not get going	1166 (9.41)	1.22 (1.04–1.43)	1.21 (0.97–1.50)

OR, odds ratio; CI, confidence interval.

a Model was adjusted for age, gender, residence, marital status, educational level, smoking status, drinking status, body pains, history of hypertension, diabetes, chronic lung diseases, heart diseases, kidney diseases, neuropsychiatric diseases and arthritis or rheumatism; and use hypertension medications, diabetes medications, heart medications, neuropsychiatric medications; and systolic blood pressure, diastolic blood pressure, pulse, body mass index, waist circumference, handgrip strength and peak expiratory flow.

b Sum of Specific Depressive Symptoms.

c Measured by the 10-item Center for Epidemiologic Studies Depression Scale (Having specific depressive symptoms were identified by defining item responses as 3–4 days or 5–7 days).

### Subgroup and sensitivity analyses

**Figure 3:**
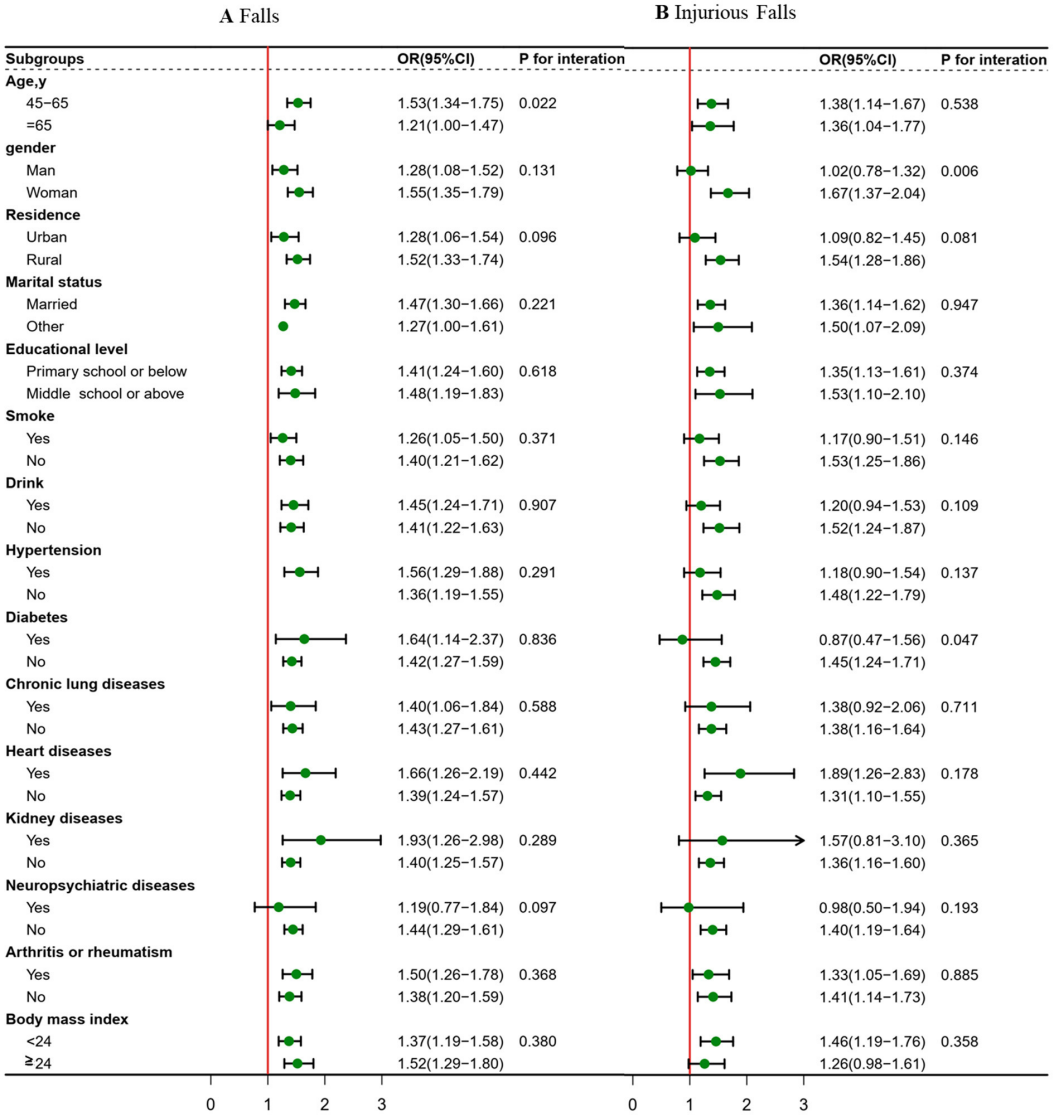
Association between depressive symptoms (high vs. low) and falls and injurious falls risk stratified by different factors. Graphs show odds ratios (OR) and 95% confidence intervals (CI) for falls **(A)** and injurious falls **(B)** adjusted for age, gender, residence, marital status, educational level, smoking status, drinking status; and history of hypertension, diabetes, chronic lung diseases, heart diseases, kidney diseases, neuropsychiatric diseases and arthritis or rheumatism; and systolic blood pressure, diastolic blood pressure, pulse, body mass index, waist circumference, handgrip strength and peak expiratory flow.

Subgroup analyses were conducted to stratify associations between depressive symptoms (high vs. low) and falls and injurious falls by risk factors (Figure 3). The effects of depressive symptoms on falls were more pronounced in participants aged 45–65 years (*p* = 0.022 for interaction), and the association between depressive symptoms and injurious falls was stronger in female participants (*p* = 0.006 for interaction) and non-diabetic participants (*p* = 0.047 for interaction). The effects of depressive symptoms on falls and injurious falls remained after further adjustment for laboratory indicators in 8.538 participants with blood sample data (Supplementary Table 2), and in repeat analyses in 9,526 participants with non-imputed data (Supplementary Table 3).

## Discussion

In the current study depressive symptoms were positively associated with falls and injurious falls during a 3-year follow-up period in middle-aged and older adults. Specifically, high depressive symptoms at baseline increased the risks of falls and injurious falls. Most specific depressive symptoms were independently associated with falls, and four specific symptoms (“had trouble concentrating”, “felt depressed”, “everything was an effort”, and “restless sleep”) were significantly associated with injurious falls.

The population selected for the study was individuals without a history of falls within the previous 2 years, based on the possibility that falls themselves may lead to depressive symptoms (17, 18). The results of the present study corroborate prior studies with regard to positive associations between high depressive symptoms and falls and injurious falls. Patients with depressive symptoms had a nearly three times greater risk of falling than those without depressive symptoms (19). In another longitudinal survey high depressive symptoms estimated by the Center for Epidemiological Studies Depression Scale 8-item short-form were associated with an increased risk of falls 2 years later (20). Systematic reviews (10, 11) also indicate relationships between high depressive symptoms and increased risks of falls in elderly adults. In the current study high depressive symptoms were a significant predictor of injurious falls, which is consistent with a prior study (7). Compared with these previous studies, this study not only had much larger sample size over a wider age range (≥45 years old at baseline) and a longer follow-up time, but also paid attention to the influence of specific depressive symptoms on falls and injurious falls.

The effects of specific depressive symptoms on falls and injurious falls have not been fully investigated. In a cross-sectional investigation attention interference and loss of executive control were associated with falls in elderly adults (21). Poor attention during gait increased the risk of falls in elderly people (22). Australian assisted-living-care residents with < 6 h sleep had a higher likelihood of experiencing multiple falls after 1 year (23). Poor sleep quality, insomnia, and increased sleep disturbances were associated with increased likelihood of recurrent falls (24). A fear of falling that reduces activity and further decreases gait quantity and quality increases the likelihood of falling (25). In a longitudinal study perceived social isolation was reportedly related to a 33% increase in fall risk (26). The present study is the first to investigate the influences of specific depressive symptoms as determined *via* a depression rating scale on falls and injurious falls.

There are several possibilities with respect to mechanisms underlying associations between depressive symptoms and falls and injurious falls. Numerous risk factors for falls such as psychomotor retardation and gait alteration are common in older people with depressive symptoms in epidemiological investigations (27, 28). Individuals with depressive symptoms also tend to exhibit cognitive deficits that affect attention, executive functions, and processing speed, which can all increase the risk of falls and injurious falls (29). By influencing levels of inflammatory cytokines, vitamin D levels, and some hormone concentrations, depressive symptoms may lead to factures (29, 30). Lastly, multiple somatic comorbidities related to falls are often associated with depression (31). Women are more likely to experience depressive symptoms than men (32), which may be the reason why depressive symptoms is more strongly associated with injurious falls in women. Prior studies supported that people with diabetes were at a significantly higher risk of falls and injurious falls than people without diabetes across (33, 34). This study found stronger associations of depressive symptoms with injurious falls among non-diabetes participants and possible reason was selection bias.

The strengths of the current study include the evaluation of specific depressive symptoms, its prospective design, inclusion of the outcomes of injurious falls, the long follow-up period, and adjustments for various confounding factors. The study also had some limitations. First, data pertaining to falls and chronic comorbidities were self- reported, which may have resulted in bias. Second, the CESD-10 was used to evaluate depressive symptoms rather than clinical diagnosis. However, our findings do highlight the importance of assessing depressive symptoms to avoid falls and injurious falls in adults aged ≥45 years. Additional specific depressive symptoms associated with falls and injurious falls may be discovered *via* the use of other validated rating scales. Third, the findings may not fully apply to other countries because the study was conducted in Chinese adults aged 45 years and over. Fourth, the study bears the characteristic limitations of *post-hoc* subgroup analysis. Despite adjustments for many confounding factors, residual confounding factors may still exist. Fifth, the falls or injuries cases within the first follow-up year should be excluded in the sensitivity analysis to exclude an inverse causal relationship. It was difficult to conduct limited by the availability of data.

## Conclusion

In the current study middle and older aged adults with high depressive symptoms were at increased risk of falls and injurious falls, and the presence of specific depressive symptoms increased the risk of injurious falls. These results are significant from a public point of view in the light of population aging. We should not only pay attention to whether or not have depressive symptoms, but also need to pay attention to the specific symptoms of depression such as “had trouble concentrating”, “felt depressed”, “everything was an effort”, and “restless sleep”. Tailored fall- prevention programs should be established for adults with specific depressive symptoms related to injurious falls.

## Data availability statement

The raw data supporting the conclusions of this article will be made available by the authors, without undue reservation.

## Author contributions

QZ and HB: designed and conducted the study. HB, SL, and DJ: statistical analysis. HB, LW, and ZT: drafting of the manuscript. HB, LW, ZT, and YD: administrative, technical, or material support. All authors read and approved the final manuscript.

## Conflict of interest

The authors declare that the research was conducted in the absence of any commercial or financial relationships that could be construed as a potential conflict of interest.

## Publisher's note

All claims expressed in this article are solely those of the authors and do not necessarily represent those of their affiliated organizations, or those of the publisher, the editors and the reviewers. Any product that may be evaluated in this article, or claim that may be made by its manufacturer, is not guaranteed or endorsed by the publisher.
