# Supplementary material for: Depressive symptoms increase the risk of falls and injurious falls in Chinese adults aged ≥ 45 years: A 3-year cohort study
Source: Front Public Health. 2022 Oct 13;10:964408. doi: 10.3389/fpubh.2022.964408 (PMC9606331; doi:10.3389/fpubh.2022.964408)
Supplement: Supplementary file 1 [file Data_Sheet_1.docx]

| **Supplemental Table1. Baseline Characteristics between Participants Excluded and Included** | | | |
| --- | --- | --- | --- |
| **Characteristics** | **Excluded** | **Included** | ***P*. Value** |
| No. of participants | 8575 | 12392 |  |
| Age, y | 59.00(49.00, 68.00) | 58.00 (51.00, 65.00) | 0.481 |
| Men | 3739(43.61) | 6266 (50.56) | <0.001 |
| Rural residence | 4911 (57.27) | 7578 (61.15) | <0.001 |
| Married | 6541 (76.28) | 10367 (83.66) | <0.001 |
| Educational Level |  |  | <0.001 |
| No formal education | 3804 (44.44) | 4675 (37.73) |  |
| Primary school | 2685 (31.37) | 3577 (28.87) |  |
| Middle or high school | 1724 (20.14) | 3646 (29.42) |  |
| College or above | 347 (4.05) | 494 (3.99) |  |
| Smoke | 3465 (40.57) | 5571 (44.96) | <0.001 |
| Drink | 3850 (45.21) | 5826 (47.05) | 0.009 |
| Body pains | 3665(36.49) | 3178 (25.65) | <0.001 |
| History of comorbidities |  |  |  |
| Hypertension | 2613 (30.95) | 3754 (30.45) | 0.455 |
| Diabetes | 741 (8.77) | 973 (7.91) | 0.029 |
| Chronic lung diseases | 1257 (14.84) | 1524 (12.36) | <0.001 |
| Heart diseases | 1295 (15.31) | 1661 (13.48) | <0.001 |
| Kidney diseases | 684 (8.09) | 714 (5.80) | <0.001 |
| Neuropsychiatric diseases | 805 (9.48) | 558 (4.52) | <0.001 |
| Arthritis or rheumatism | 2973 (35.03) | 3897 (31.58) | <0.001 |
| History of medication use |  |  |  |
| Hypertension medications | 1979 (23.51) | 2806 (22.92) | 0.332 |
| Diabetes medications | 521 (6.17) | 675 (5.49) | 0.042 |
| Heart medications | 902 (10.68) | 1069 (8.69) | <0.001 |
| Neuropsychiatric medications | 406 (4.95) | 250 (2.06) | <0.001 |
| Systolic blood pressure, mmHg | 126.82 (20.60) | 127.00 (19.21) | 0.583 |
| Diastolic blood pressure, mmHg | 74.52 (11.72) | 75.15 (11.14) | <0.001 |
| Pulse, /min | 74.61 (10.49) | 73.83 (10.46) | <0.001 |
| Body mass index, kg/m^2^ | 23.65 (3.69) | 24.01 (3.64) | <0.001 |
| Waist circumference, cm | 84.85 (12.94) | 85.79 (12.69) | <0.001 |
| Handgrip strength, kg | 28.19 (10.91) | 30.98 (10.50) | <0.001 |
| Peak expiratory flow, l/min | 300.17 (123.77) | 327.79 (122.77) | <0.001 |
| 1.Data are shown as means ± standard deviation, median (interquartile range), or numbers (percentages).  2. Missing data: 91 (0.43 %) for age, 2 (0.01 %) for gender, 15 (0.07 %) for education level, 35 (0.17 %) for smoke, 68 (0.32 %) for drink, 1275 (6.08 %) for body pains, 194 (0.93 %) for hypertension, 223 (1.06 %) for diabetes, 168 (0.80 %) for chronic lung diseases, 188 (0.90 %) for heart diseases, 194 (0.93 %) for kidney diseases, 136 (0.65 %) for neuropsychiatric diseases, 140 (0.67 %) for arthritis or rheumatism, 311 (1.48 %) for hypertension medications, 240 (1.14 %) for diabetes medications, 214 (1.02 %) for heart medications, 643 (3.07 %) for neuropsychiatric medications, 4789 (22.84 %) for systolic blood pressure, 4792 (22.85 %) for diastolic blood pressure, 4798 (22..88 %) for pulse, 4778 (22.79 %) for body mass index, 4728 (22.55 %) for waist circumference,4879 (23.27 %) for handgrip strength and 4948 (23.60 %) for peak expiratory flow. | | | |

| **Supplemental Table2. Association of Depressive Symptoms (high versus low) with Falls and Injurious Falls in Subpopulations of 8487 Participants Provided Blood Samples** | | |
| --- | --- | --- |
|  | **OR (95%CI)** |  |
|  | **Falls** | **Injurious Falls** |
| Model 4^a^ | 1.37(1.20-1.56) | 1.36(1.13-1.64) |
| Model adjusted as model 4 puls |  |  |
| White Blood Cell | 1.37(1.21-1.56) | 1.36(1.13-1.64) |
| Hemoglobin | 1.37(1.21-1.56) | 1.36(1.13-1.64) |
| Platelet | 1.37(1.21-1.56) | 1.36(1.13-1.64) |
| C-reactive protein | 1.37(1.21-1.56) | 1.36(1.13-1.64) |
| Estimated glomerular filtration rate | 1.37(1.21-1.56) | 1.36(1.13-1.64) |
| Glucose | 1.37(1.20-1.56) | 1.36(1.13-1.64) |
| Uric acid | 1.37(1.21-1.57) | 1.37(1.13-1.64) |
| All laboratory indicators | 1.38(1.21-1.58) | 1.38(1.14-1.66) |
| Abbreviation: OR, odds ratio; CI, confidence interval ^a^ Model 4 was adjusted for age, gender, residence, marital status, educational level, smoking status, drinking status, body pains; and history of hypertension, diabetes, chronic lung diseases, heart diseases, kidney diseases, neuropsychiatric diseases and arthritis or rheumatism; and use hypertension medications, diabetes medications, heart medications, neuropsychiatric medications; and systolic blood pressure, diastolic blood pressure, pulse, body mass index, waist circumference, handgrip strength and peak expiratory flow. | | |

| **Supplemental Table3. Association of Depressive Symptoms with Falls and Injurious Falls in Subpopulations of 9526 Participants with Non-imputed Data** | | | | |
| --- | --- | --- | --- | --- |
|  | **OR (95%CI)** | | | |
| **Outcome** | **Model 1^a^** | **Model 2^b^** | **Model 3^c^** | **Model 4^d^** |
| Falls |  |  |  |  |
| Categories |  |  |  |  |
| Low, <10 | 1[Reference] | 1[Reference] | 1[Reference] | 1[Reference] |
| High, ≥10 | 1.53(1.36-1.72) | 1.49(1.32-1.68) | 1.30(1.14-1.48) | 1.28(1.12-1.45) |
| Injurious Falls |  |  |  |  |
| Categories |  |  |  |  |
| Low, <10 | 1[Reference] | 1[Reference] | 1[Reference] | 1[Reference] |
| High, ≥10 | 1.57(1.32-1.85) | 1.51(1.27-1.79) | 1.34(1.11-1.60) | 1.30(1.08-1.57) |
| Abbreviation: OR, odds ratio; CI, confidence interval ^a^ Model 1 was adjusted for age and gender. ^b^ Model 2 was adjusted for age, gender, residence, marital status, educational level, smoking status, drinking status. ^c^ Model 3 was adjusted as model 2 plus body pains, history of hypertension, diabetes, chronic lung diseases, heart diseases, kidney diseases, neuropsychiatric diseases and arthritis or rheumatism; and use hypertension medications, diabetes medications, heart medications, neuropsychiatric medications. ^d^ Model 4 was adjusted as model 3 plus systolic blood pressure, diastolic blood pressure, pulse, body mass index, waist circumference, handgrip strength and peak expiratory flow. | | | | |
